# Secretory RAB GTPase 3C modulates IL6-STAT3 pathway to promote colon cancer metastasis and is associated with poor prognosis

**DOI:** 10.1186/s12943-017-0687-7

**Published:** 2017-08-07

**Authors:** Yu-Chan Chang, Chia-Yi Su, Ming-Huang Chen, Wei-Shone Chen, Chi-Long Chen, Michael Hsiao

**Affiliations:** 1 Graduate Institute of Life Sciences, National Defense Medical Center, Taipei, Taiwan; 2 Genomics Research Center, Academia Sinica, Taipei, Taiwan; 3 Department of Oncology, Taipei Veterans General Hospital, Taipei, Taiwan; 4 School of Medicine, National Yang-Ming University, Taipei, 112 Taiwan; 5 Division of Colon & Rectal Surgery, Department of Surgery, Taipei Veterans General Hospital, Taipei, Taiwan; 6 Department of Surgery, Faculty of Medicine, School of Medicine, National Yang-Ming University, Taipei, Taiwan; 7 Department of Pathology, Taipei Medical University Hospital, Taipei Medical University, Taipei, Taiwan; 8 Department of Pathology, College of Medicine, Taipei Medical University, Taipei, Taiwan; 9 Department of Biochemistry, College of Medicine, Kaohsiung Medical University, Kaohsiung, Taiwan

**Keywords:** RAB GTPases, RAB3C, IL-6, STAT3, Colon cancer

## Abstract

**Background:**

RAB GTPases are important in the regulation of membrane trafficking and cell movement. Recently, exocytic RABs have received increasing attention in cancer research. However, the functional roles of exocytic RABs in colorectal carcinogenesis remain to be elucidated.

**Methods:**

Immunohistochemistry analysis of a microarray containing 215 colorectal adenocarcinoma tissues was used to identify the association between exocytic RABs and patient prognosis. Complementary functional RAB3C overexpression and knockdown experiments were performed. The molecular mechanism of RAB3C in inducing colon cancer cell metastasis was determined.

**Results:**

High RAB3C expression in patients was found to be significantly associated with advanced pathological stage, distant metastasis and poor prognosis. Multivariate analyses showed that high RAB3C expression was an independent prognostic marker in overall (*P* = 0.001) and disease-free survival (*P* < 0.001). Furthermore, our experimental results showed an increase in the migration and invasion ability of RAB3C-overexpressing colon cancer cells and increased metastatic nodules in a mouse metastasis model. The effect of RAB3C-overexpressing cell-conditioned medium was found to significantly promote the migration ability of parental colon cancer cells, thus suggesting that the promotion of migration is exocytosis dependent. Upregulation of other exocytic RABs was also seen in RAB3C-overexpressing cells. Through microarray and proteomics analyses, increased production of multiple cytokines was observed in RAB3C-overexpressing cell lines, and the IL-6 pathway was the top pathway whose members exhibited gene expression changes after RAB3C overexpression, according to Ingenuity Pathway Analysis. Blocking IL-6 with IL-6 antibody treatment or IL-6 knockdown significantly inhibited the migration potential of RAB3C-overexpressing colon cancer cells. In addition, IL-6 was found to induce STAT3 phosphorylation in RAB3C-overexpressing colon cancer cells, thus promoting migration. Ruxolitinib, a JAK2 inhibitor, was found to significantly inhibit RAB3C-induced colon cancer cell migration.

**Conclusions:**

Our study revealed that RAB3C overexpression promotes tumor metastasis and is associated with poor prognosis in colorectal cancer, through modulating the ability of cancer cells to release IL-6 through exocytosis and activate the JAK2-STAT3 signaling pathway. These results further suggest that inhibition of STAT3 phosphorylation in the RAB3C-IL-6-STAT3 axis by using Ruxolitinib may be a new therapeutic strategy to combat metastatic colon cancers.

**Electronic supplementary material:**

The online version of this article (doi:10.1186/s12943-017-0687-7) contains supplementary material, which is available to authorized users.

## Background

Colorectal cancer is one of the most common cancers in the world, and is associated with a high death rate [1]. Moreover, an apparent increase in the incidence of this cancer has been reported in many Asian countries [2]. In spite of major advances in screening and treatment, the overall survival rates are still low in patients diagnosed in late stages of the disease. Distant metastasis causes most of the cancer-related morbidity and mortality after initial treatment. However, targeted treatment, such as anti-epidermal growth factor receptor (EGFR) and anti-vascular endothelial growth factor (VEGF) therapies, have had a relatively minor effect on the survival of metastatic colorectal cancer patients [3]. Unraveling the mechanism of tumor progression and further discovery of novel prognostic markers for prediction and treatment evaluation is urgently required.

RAB GTPases, a large family of Ras small GTPases, play crucial roles in normal human physiology by regulating membrane identity and vesicle trafficking, including budding, sorting, uncoating, motility, tethering, and fusion [4]. Loss of RAB GTPase activity is known to cause many inherited disorders [5]. Neurons, which are connected through synapses, are vulnerable to dysfunction of RAB-regulated membrane trafficking. RAB GTPases have been reported to be involved in the pathogenesis of Parkinson’s disease and Huntington’s disease [6–8]. RABs also have a role in the pathogenesis of type II diabetes through regulation of glucose transporter GLUT4 translocation [9]. In addition, RABs also participate in phagocytosis of many pathogens, such as intracellular bacteria and viruses, and are crucial mediators of the innate immune response against infection [10, 11].

Although the role of the Ras proto-oncogene in tumorigenesis has been well studied, the importance of RAB GTPases in cancer remains largely unknown. Among all the RABs, endocytic RABs, such as RAB5, RAB21, and RAB25, are the most extensively studied. The well-characterized example is RAB25, which modulates the movement of integrin-recycling vesicles [12]. Several lines of evidence have indicated that RAB25 has a large impact on epithelial cell transformation, tumor motility and the invasive ability of several epithelial cancers [13–15]. Overexpression of RAB25 is associated with aggressive behavior in breast cancer and ovarian cancer [16, 17]. However, an opposite effect has also been reported, in which RAB25 acts as a tumor suppressor in colon cancer [14]. RAB5, which is essential for the fusion of early endosomes, is another intensively studied endocytic RAB in cancer. RAB5 regulates cell survival and migration through caspase-8-mediated signal transduction [18]. However, several studies have noted that RAB5 expression appears to be decreased during cell migration [19–21]. Both overexpression and downregulation of RAB5 have been reported in different cancers [22, 23].

Ras-related protein 3C (RAB3C), a secretory RAB, participates in the modulation of secretory vesicle exocytosis [5]. The secretory RABs, which include RAB3, RAB26, RAB27, and RAB37, have effects on carcinogenesis similar to those of endocytic RABs. Recent studies have shown that RAB27A and RAB27B are associated with invasiveness and metastasis in cancer [24, 25]. However, there has been far less research on the correlation between cancer and RAB3. RAB3 consists of 4 isoforms, RAB3A, RAB3B, RAB3C, and RAB3D. The estimated protein expression from the Genecard website indicates that RAB3A and RAB3B are predominantly expressed in neural systems and neuroendocrine cells, whereas RAB3D is primarily present in nonneural tissues [26–28]. RAB3C is normally expressed in peripheral blood mononuclear cells and platelets, and in nervous system, colon, ovary and seminal vesicles. However, the function of RAB3C is relatively unclear, and an association between RAB3C and cancer has yet to be determined. In addition, in recent years, increased attention has been paid to the role of RAB-regulated exosome secretion in the progression of various cancers, including colon cancer [29, 30]. Therefore, in this study, we investigated the role of RAB3C in colon carcinogenesis. Our immunohistochemistry analysis results showed that high RAB3C expression was significantly correlated with poor prognosis and more frequent distant metastasis in clinical colorectal cancer patients. Increases in migration and invasion ability in vitro and the metastasis-promoting ability in vivo were found after RAB3C overexpression in colon cancer cells. The dose-dependent decrease in the migration-enhancing ability of RAB3C-overexpressing cell-conditioned medium after IL-6 blockade further confirmed the results of both RNA microarray and proteomics analyses, which showed that the IL-6-STAT3 signaling axis is the top ranking activated pathway. Collectively, RAB3C upregulation facilitates colorectal cancer metastasis by promoting IL-6 secretion and recruiting members of the STAT3-related pathway. Therefore, targeting the RAB3C-IL-6-STAT3 axis by using therapeutics, such as Ruxolitinib, might be a useful strategy to combat metastatic colon cancers.

## Methods

### Patients

In total, 215 patients diagnosed with colorectal adenocarcinoma at the Taipei Municipal Wan Fang Hospital of Taiwan from 1998 to 2005 were included in this study. Patients who received preoperative chemotherapy or radiation therapy or who received incomplete surgical resection were excluded. All cases were staged according to the 7th version of the cancer staging manual of the American Joint Committee on Cancer, and the histological cancer type was classified according to the World Health Organization classification. Follow-up data were available in all cases, and the last clinical follow-up time was January 2011. Overall survival (OS) and disease-free survival (DFS) were defined as the interval from surgery to death from any cause and recurrence or distant metastasis or death, respectively.

### Tissue microarray construction and immunohistochemistry staining

Paraffin-embedded tissues used to generate tissue microarrays were collected from Taipei Medical University Hospital with IRB approval (TMU-IRB 99049). Written informed consent was obtained from each patient included in the study. Three representative 1-mm-diameter cores from each tumor taken from the formalin-fixed paraffin-embedded tissues were selected on the basis of morphology typical of the diagnosis. Assessable cores were obtained in a total of 215 cases. Moreover, paired normal mucosal tissues were also obtained in 62 of the 215 cases. The histopathological diagnoses of all samples were reviewed and confirmed by two pathologists via hematoxylin- and eosin-stained slides. Immunohistochemistry staining was performed on serial 5-μm-thick tissue sections cut from the tissue microarray (TMA) by using an automated immunostainer (Ventana Discovery XT autostainer, Ventana Medical Systems, Tucson, AZ). Briefly, sections were dewaxed in a 60 °C oven, deparaffinized in xylene, and rehydrated in graded alcohol. Antigens were retrieved by heat-induced antigen retrieval for 30 min with TRIS-EDTA buffer. Slides were stained with a polyclonal rabbit anti-human RAB3A antibody (15029–1-AP, 1:200; Proteintech, Chicago, USA), a polyclonal rabbit anti-human RAB3B antibody (GTX104360, 1:100; GeneTex, Taipei, Taiwan), a polyclonal rabbit anti-human RAB3C antibody (GTX108610, 1:500; GeneTex, Taipei, Taiwan), a polyclonal rabbit anti-human RAB3D antibody (12320–1-AP, 1:50; Proteintech, Chicago, USA), a polyclonal rabbit anti-human RAB26 antibody (GTX118872, 1:100; GeneTex, Taipei, Taiwan), a polyclonal rabbit anti-human RAB27A antibody (GTX109180, 1:750; GeneTex, Taipei, Taiwan), a polyclonal rabbit anti-human RAB27B antibody (13412–1-AP, 1:200; Proteintech, Chicago, USA), and a polyclonal rabbit anti-human RAB37 antibody (13051–1-AP, 1:100; Proteintech, Chicago, USA). The sections were subsequently counterstained with hematoxylin, dehydrated, and mounted.

### TMA immunohistochemistry interpretation

The IHC staining assessment was independently conducted by 2 pathologists who were blinded to patient outcome. Only cytoplasmic expression of tumor cells in the cores were evaluated. Both the immunoreactivity intensity and the percentage were recorded. The intensity of staining was scored using a four-tier scale and defined as follows: 0, no staining; 1+, weak staining; 2+, moderate staining; 3+, strong staining. The extent of staining was scored by the percentage of positive cells (0–100%). The final IHC scores (0–300) were obtained by multiplying the staining intensity score by the percentage of positive cells. All cases were divided into two groups according to the final IHC scores. High IHC expression level was defined as a score greater than or equal to 150, and a score less than 150 was defined as low expression.

### Cell culture

Eight human colon cancer cell lines of which three cell lines (CX-1, DLD-1, H3347) were maintained in RPMI 1640 medium and two cell lines (HCT116 and HT-29) were maintained in McCoy’s 5A modified medium (Sigma, St. Louis, MO, USA). Mediums were all supplemented with 10% fetal bovine serum (GIBCO, Grand Island, NY, USA), penicillin (100 unit/ml), and streptomycin (100 μg/ml). Cells were incubated in 95% air, 5% CO_2_ humidified atmosphere at 37 °C. SW48, SW480, and SW620 cells were cultured in Leibovitz L-15 medium (Sigma, St. Louis, MO, USA) and incubated in CO_2_ free incubator.

### Western blot analysis

The cells were lysed at 4 °C in RIPA buffer supplemented with protease and phosphatase inhibitors. Equal amounts (30 μg) of protein were separated electrophoretically using SDS-polyacrylamide gels and then transferred to PVDF membranes (Millipore, Bedford, MA, USA). After being blocked with 5% non-fat milk, the membrane was incubated with specific antibodies (RAB3C: GTX108610, 1:5000, GeneTex, Taipei, Taiwan; RAB27A: GTX109180, 1:5000, GeneTex, Taipei, Taiwan; RAB3B: GTX108610, 1:5000; GeneTex, Taipei, Taiwan; RAB26: GTX118872, 1:5000; GeneTex, Taipei, Taiwan; IL-6: GTX110527, 1:1000, GeneTex, Taipei, Taiwan; STAT3: #4904, 1:1000, Cell Signaling, USA; phospho-STAT3: #9145, 1:1000, Cell Signaling, USA) overnight at 4 °C and then incubated with horseradish peroxidase-conjugated secondary antibody for 1 h. The blots were visualized using an ECL-Plus detection kit (PerkinElmer Life Sciences, Boston, MA, USA).

### Virus production and Infection

Full length RAB3C cDNAs were amplified from the MGC gene bank (Open Biosystem Inc., from Dr. Michael Hsiao’s library) by using PCR. The cDNAs were first cloned into a pENTR1A vector (Gateway pENTR 1A Dual Selection Vector), then subcloned into pLenti6.3/V5-DEST. Target cells were seeded at the appropriate density in a 6-well dish 24 h prior to infection. On the day of infection, the growth medium was removed, and 1500 μl of medium containing lentivirus and polybrene (final concentration 8 μg/ml) was added to each well of the 6-well plate. The plate was then centrifuged for 1 h at 37 °C and 1200 g. Subsequently, the plate was incubated for 24 h. After incubation, the medium was removed, and fresh medium containing blasticidin was added. Then, 72 h after infection, the cells were further split, and the selection was continued until all of the control cells were dead.

### Migration and invasion assay

Migration and invasion abilities of cells were evaluated by transwell assay (Millipore, Bedford, MA, USA). For invasion assay, transwells were pre-coated with 35 μl of 3X diluted matrix matrigel (Bd Biosciences Pharmingen, San Diego, CA, USA) for 30 min. The upper chamber of the device were added with 2 × 10^5^ cells in serum-free culture medium, and the lower chamber was filled with 10% FBS culture medium. After indicated hours of incubation, the remaining cells on the upper surface of the filter were carefully removed with a cotton swab. The filter was then fixed, stained and photographed. Migrated or invaded cells were quantified by counting the cells in three random fields per filter.

### Animal study

All animal experiments were conducted in accordance with a protocol approved by the Academia Sinica Institutional Animal Care and Utilization Committee. Age-matched male NSG mice (6–8 weeks of age) were used. To evaluate metastasis status, 1 × 10^6^ cells were resuspended in 0.1 ml of PBS and injected into the lateral tail vein (*n* = 6). Metastatic lung nodules were counted and were further confirmed via H&E staining under a microscope.

### cDNA microarray and data analysis

Total RNA extracted from cells with a A260/280 ratio greater than 1.9 was used in the Affymetrix cDNA microarray analysis. In the analysis, hybridization was performed with Affymetrix human U133 2.0 plus arrays, and the chips were scanned with an Affymetrix GeneChip scanner 3000. Then, Affymetrix DAT files were processed by an Affymetrix Gene Chip Operating System (GCOS) to generate CEL files. The raw intensities in the CEL files were normalized by robust multi-chip analysis, and a fold-change analysis was performed using GeneSpring GX11 (Agilent Technologies).

### Proteomics

A non-labeling quantification method was used to analyze our proteomic data. Briefly, 20 μg of protein from each sample was loaded on SDS-PAGE gels for separation. After electrophoresis, the protein was detected with Coomassie blue staining. Each lane of the SDS-PAGE gels was cut into 10 pieces, with a similar amount of protein in each piece. Then, every piece was processed by in-gel digestion with trypsin to produce a large number of peptide fragments. These fragments were detected and measured by LTQ-FT (Thermo) at the proteomics core facility at the Genomics Research Center, Academia Sinica. Data from 10 pieces of the same original sample were combined, and the protein expression was calculated by using MaxQuant (version: 1.3.0.5) and analyzed with Perseus (version: 1.3.0.4).

### In silico analysis

Gene expression levels were normalized as log2 values in GeneSpring software (Agilent Technologies, Palo Alto, CA, USA). Genes that were up or downregulated with a greater than 2.0-fold change in the RAB3C overexpression group compared with the vector control group were collected. We further performed computational simulation by using Ingenuity Pathway Analysis (IPA; QIAGEN, Valencia, CA, USA) online tools to predict potential upstream regulators and canonical pathways. Pathway analysis was performed according to the genes and proteins with over 2.0-fold change in expression and an activation z-score of over 2.0 compared with vector control cells identified from the microarray and proteomics data, respectively.

### Statistical analysis

Statistical analysis was performed with SPSS 20 software (SPSS, Chicago, Illinois, USA). A paired t-test was performed to compare the RAB3C IHC expression in cancer tissue and the corresponding normal mucosal tissue. The associations between clinicopathological categorical variables and RAB3C IHC expression were assessed by Pearson’s chi-square test. Survival rates were analyzed by using the Kaplan-Meier method and were compared with a log-rank test. Univariate and multivariate analysis were performed using Cox proportional hazards regression analysis without and with an adjustment for RAB3C IHC expression level and various clinicopathological parameters. For all clinical analyses, a *P* value of <0.05 was considered statistically significant. Student’s *t*-test was also used to compare the results of migration and invasion assays and *in vivo* metastasis experiments. The *P* values with the following levels were considered significant: *P < 0.05, **P < 0.01, and ***P < 0.001.

## Results

### High RAB3C expression is an independent indicator of poor prognosis for colorectal cancer patients

**Fig. 1:**
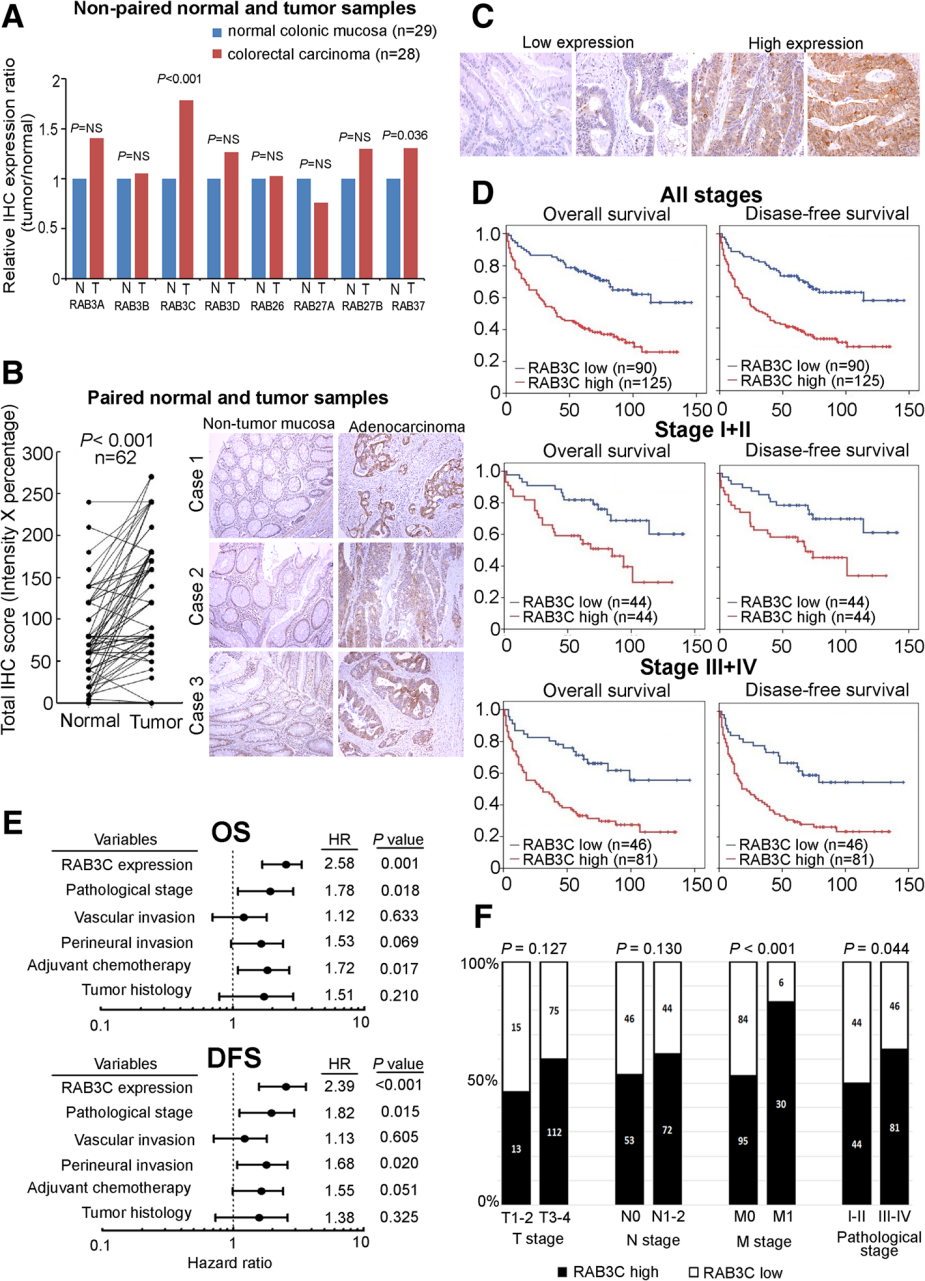
High RAB3C expression is an independent indicator of poor prognosis, distant metastasis and advanced stage in colorectal cancer patients. **a** Screening of exocytic RAB expression levels using immunohistochemistry (IHC) staining in colorectal cancer identified significant RAB3C overexpression in colorectal cancer tissue compared with normal colonic mucosa. **b** Increased RAB3C expression was also confirmed through comparison of paired normal and colorectal cancer tissue samples. The scores were calculated as the staining intensity score × the percentage of stained cells. Images were taken at a magnification of 200×. Scale bars represent 100 μm. **c** Negative and weak cytoplasmic RAB3C IHC staining were classified as low RAB3C expression, and moderate to strong staining cytoplasmic RAB3C IHC staining were classified as high RAB3C expression. Images were taken at a magnification of 400×. Scale bars represent 200 μm. **d** For stage I to IV patients, early stage patients, and late stage patients, Kaplan-Meier plots show that patients with high RAB3C expression displayed poorer overall and disease-free survival than those with low RAB3C expression. **e** In multivariate analysis, RAB3C remained an independent prognostic factor for overall and disease-free survival. **f** High RAB3C expression indicated frequent distant metastasis and late pathological stage in the clinicopathological analysis. All cases were staged according to the 7th version of cancer staging manual of the American Joint Committee on Cancer, and the histological cancer type was classified according to World Health Organization classification

To identify which exocytic RAB has the greatest clinical importance in colorectal cancer, we screened the expression levels of all 8 exocytic RABs (RAB3A, RAB3B, RAB3C, RAB3D, RAB26, RAB27A, RAB27B and RAB37) through immunohistochemistry (IHC) staining of non-paired normal colonic mucosa and colorectal cancer samples. Significant RAB3C overexpression was identified in colorectal cancer tissue compared with normal colonic mucosa and the other 7 exocytic RABs (Fig. 1a). Then, we confirmed the finding with paired colorectal cancer tissues and corresponding normal colon mucosal tissues in 62 patients (Fig. 1b). An IHC analysis was performed to compare the RAB3C protein expression levels in both normal and cancer tissues. RAB3C immunoreactivity was significantly more intense and diffuse in tumor tissue compared with normal tissue (*P* < 0.001).

After identifying RAB3C in all 8 exocytic RABs, we further analyzed the prognostic impact of RAB3C in colorectal cancer patients. Of all the 215 colorectal cancer patients, high RAB3C expression level defined by moderate to strong cytoplasmic RAB3C IHC staining was observed in 125 (58.1%) cases, and low RAB3C expression level defined by negative or weak cytoplasmic RAB3C IHC staining was observed in 90 (41.9%) cases (Fig. 1c and d). Through a Kaplan-Meier survival analysis, high RAB3C expression in patients was found to be significantly correlated with poor OS (*P* < 0.001) and DFS (*P* = 0.001) (Fig. 1d). When patients were stratified into subgroups according to pathological stage, high RAB3C expression remained significantly correlated with poor prognosis in early stage (stage I and II) patients (*P* = 0.004 for OS and *P* = 0.008 for DFS) and in late stage (stage III and IV) patients (*P* < 0.001 for OS and *P* < 0.001 for DFS) (Fig. 1d). In a multivariate analysis, high RAB3C expression (hazard ratio [HR] = 2.58, 95% confidence interval [CI] = 1.68–3.95, *P* = 0.001 for OS; HR = 2.39, 95% CI = 1.58–3.62, *P* ≤ 0.001 for DFS) remained an independent prognostic factor for OS and DFS (Fig. 1e).

### Colorectal cancer patients with high RAB3C expression have more frequent distant metastasis and higher pathological stage

Associations between RAB3C immunoexpression and clinicopathological parameters are summarized in Additional file 1: Table S1. High RAB3C expression was positively correlated with higher M stage (*P* < 0.001), higher pathological stage (*P* = 0.044), and tumor recurrence (*P* = 0.006) (Fig. 1f and Additional file 1: Table S1). Moreover, there was a trend toward a positive correlation between high RAB3C expression and higher T stage (*P* = 0.127), lymph node metastasis (*P* = 0.130), vascular invasion (*P* = 0.107), and perineural invasion (*P* = 0.105) (Fig. 1f and Additional file 1: Table S1). To supplement our IHC analysis, we further validated our results by checking the prognostic and clinicopathological roles of RAB3C in public GSE datasets (Additional file 2: Figure S1). A high RAB3C RNA expression level was significantly correlated with poor prognosis in the GSE17536 dataset (Additional file 2: Figure S1A). In this dataset, among all 8 of the exocytic RABs, we also observed the strongest association between RAB3C and tumor grade (*P* = 0.002) (Additional file 2: Figure S1A). In another GSE dataset, GSE 21510, the strongest association was also seen between RAB3C and higher stage (*P* = 0.047) among all 8 of the exocytic RABs (Additional file 2: Figure S1B).

### RAB3C overexpression enhances the migration and invasion ability of colon cancer cells and promotes tumor metastasis in a xenograft model

**Fig. 2:**
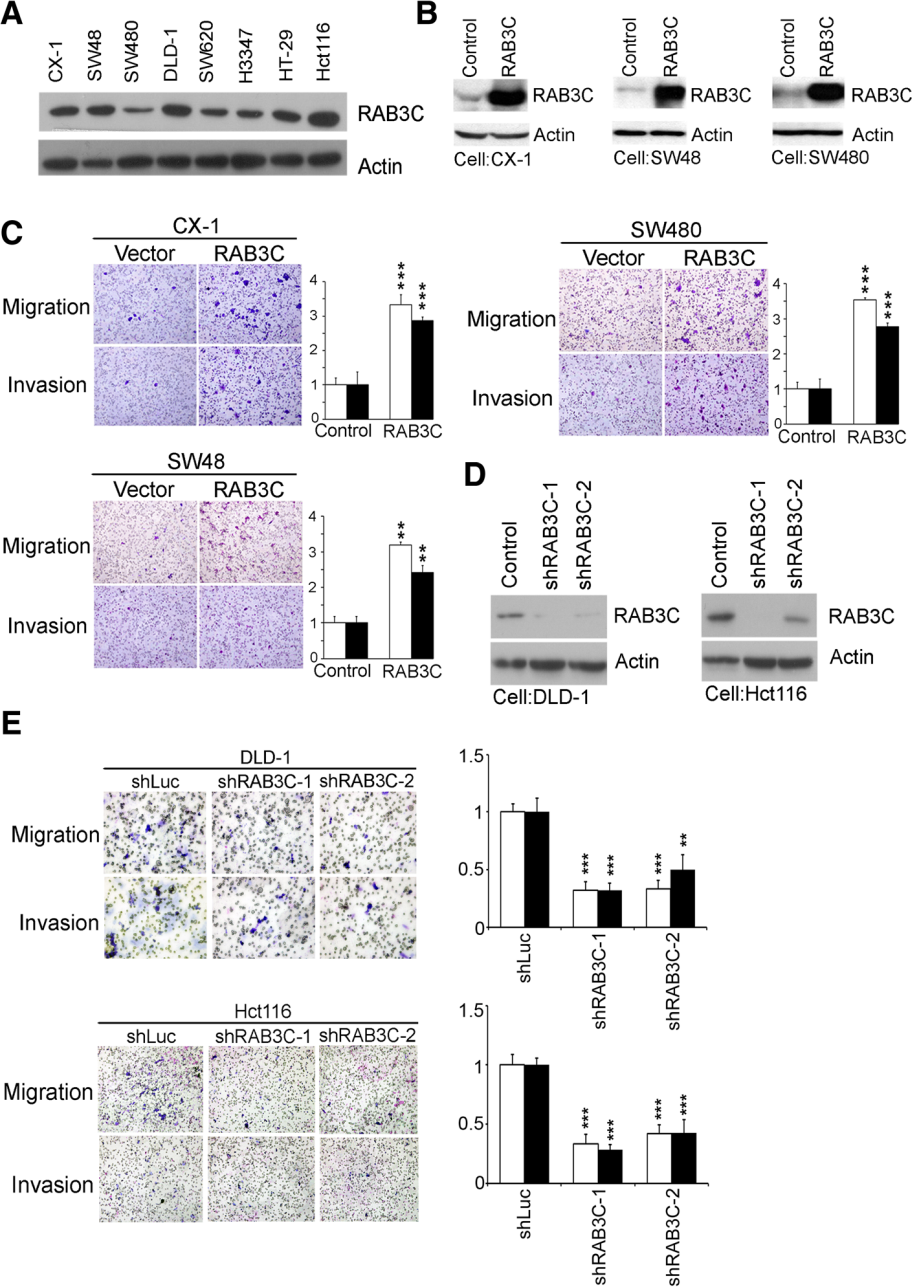
Overexpression of RAB3C enhances migration and invasion ability in vitro. **a** Variable endogenous RAB3c levels in different colon cancer cell lines. **b** Overexpression of RAB3C in CX-1, SW48, and SW480 colon cancer cell lines. **c** Overexpression of RAB3C enhanced the migration and invasion ability of colon cancer cells. **d** Knockdown of RAB3C in DLD-1 and Hct116 colon cancer cell lines. **e** Suppression of RAB3C decreased the migration and invasion ability of colon cancer cells

We detected the endogeous RAB3C protein levels in colon cancer cell panel and observed higher RAB3C expressions in more malignant cell lines (Fig. 2a, lane 4–8). Therefore, we overexpressed RAB3C in less malignant colon cancer cell lines (CX-1, SW48, and SW480) to investigate the influence of RAB3C ectopic expressions on cancer cell migration and invasion ability (Fig. 2b). In transwell migration and invasion assays, increased cell migration and invasion ability was observed in colon cancer cell lines after RAB3C overexpression (Fig. 2c). We also established a RAB3C knockdown experiment in two colon cell lines with high endogenous RAB3C expression (DLD-1 and Hct116). The results showed that decreased RAB3C expression downregulated the migration/invasion ability in colon cancer cells (Fig. 2d and e).

**Fig. 3:**
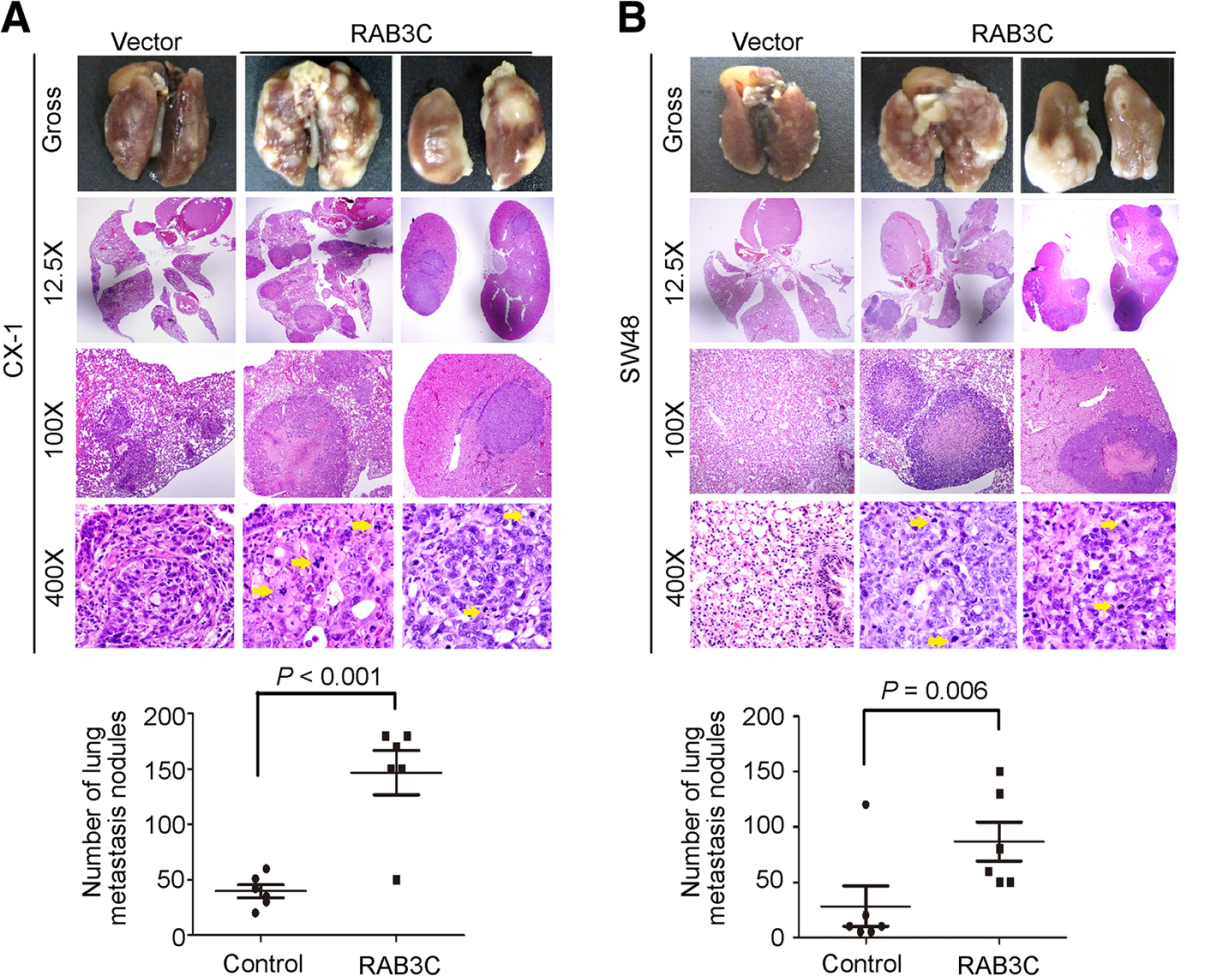
Overexpression of RAB3C increased the number of metastatic nodules in vivo (**a**-**b**) A mouse metastasis model was established by intravenous injection of RAB3C-overexpressing cells and vector control cells using (**a**) CX-1 and (**b**) SW48 cells. An increased number of metastatic nodules was observed in the lung and kidney in the RAB3C-overexpressing group compared with the control group. Some unusual mitotic figures were also observed in metastatic nodules formed from RAB3C-overexpressing cells

Metastatic animal model experiments were then performed to evaluate the tumor progression effect of RAB3C in colon cancer. CX-1 and SW48 cells with RAB3C overexpression were injected into NSG mice via the tail vein. A significant increase in the number of lung metastasis nodules was observed in the RAB3C overexpression groups compared with the corresponding vector control cell-injected groups in both CX-1 (*P* < 0.001) and SW48 cell lines (*P =* 0.006) (Fig. 3a and b). In addition to the increase in lung metastatic nodules, kidney metastasis was also seen in the RAB3C expression groups (Fig. 3a and b). In contrast, no metastatic nodules were found in the kidneys of the vector control cell-injected groups. Another interesting finding was that tumor cells with unusual mitotic figures were observed in tumor nodules of RAB3C-overexpressing cells, whereas no unusual mitotic figures were seen in vector control tumor cells (Fig. 3a and b). The above in vitro and in vivo results further confirmed the metastasis-promoting function of RAB3C in colon cancer cells.

### RAB3C overexpression induces cytokine expression, and IL-6 is the top downstream pathway with gene and protein expression upregulation

**Fig. 4:**
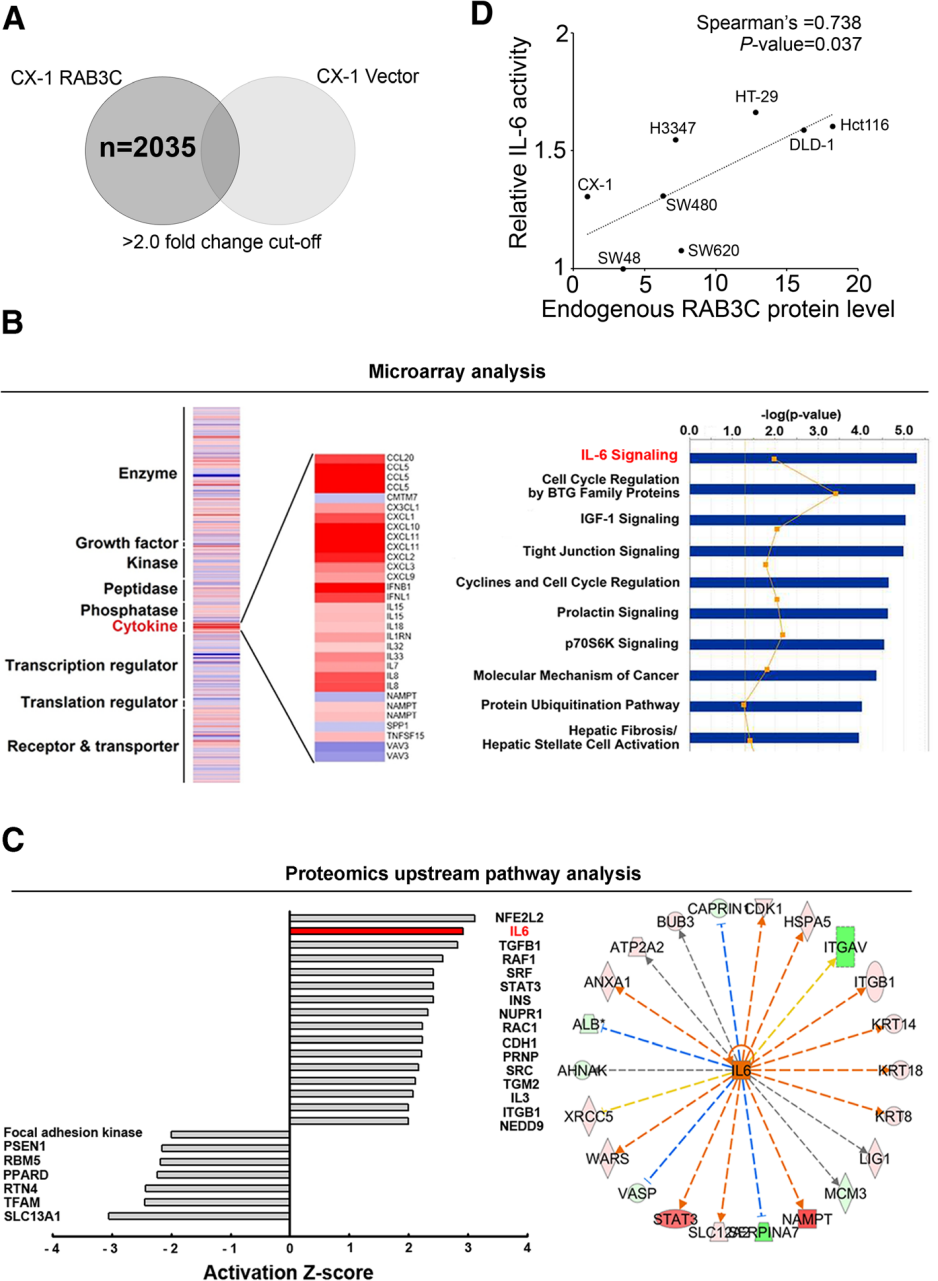
RNA microarray and pathway analyses revealed that RAB3C regulates the IL-6 signaling pathway (**a**) Putative probes regulated by RAB3C were identified from genes upregulated/downregulated at least 2-fold in CX-1 RAB3C cells compared with vector control cells. *P* < 0.05 was considered significant enrichment. **b** An RNA microarray analysis showed that RAB3C overexpression induced the expression of many cytokines. The IL-6 pathway was the top pathway hose members exhibited gene expression changes after RAB3C overexpression, according to Ingenuity Pathway Analysis (IPA). **c** IPA upstream pathway analysis of mass spectrometry proteomics data also revealed that IL-6 is a key downstream pathway in RAB3C-overexpressing cells. **d** Correlation between the endogenous RAB3C protein level and IL-6 activity in colon cancer cell lines

To determine the underlying mechanism of RAB3C-regulated tumor progression in colon cancer, mass spectrometry and a microarray analysis were performed, and the possible pathway signaling was predicted by identifying differences in the RNA and protein composition between control and RAB3C-overexpressing cells. We detected a signature by recruiting several probes with a cutoff value of >2.0-fold change in RAB3C-overexpressing CX-1 cells compared with control cells (Fig. 4a). RAB3C overexpression was found to evoke the expression of many types of cytokines in the microarray analysis (Fig. 4b). In addition, the IL-6 pathway was predicted to be the top pathway whose members exhibited gene expression changes after RAB3C overexpression, according to Ingenuity Pathway Analysis (IPA) (Fig. 4b). In an upstream pathway analysis using proteomics data, the IL-6 pathway was also one of the top activated pathways after RAB3C overexpression (Fig. 4b). IPA analysis of proteomics data focusing on IL-6 is illustrated in Fig. 4c. We thus measured the IL-6 production in the culture supernatant of each of the colon cancer cell lines. We observed that the IL-6 production was positively correlated with the endogenous RAB3C protein level in a panel of colon cancer cell lines (Fig. 4d).

### RAB3C overexpression increases exocytosis in colon cancer cells and promotes metastasis through IL-6 secretion

**Fig. 5:**
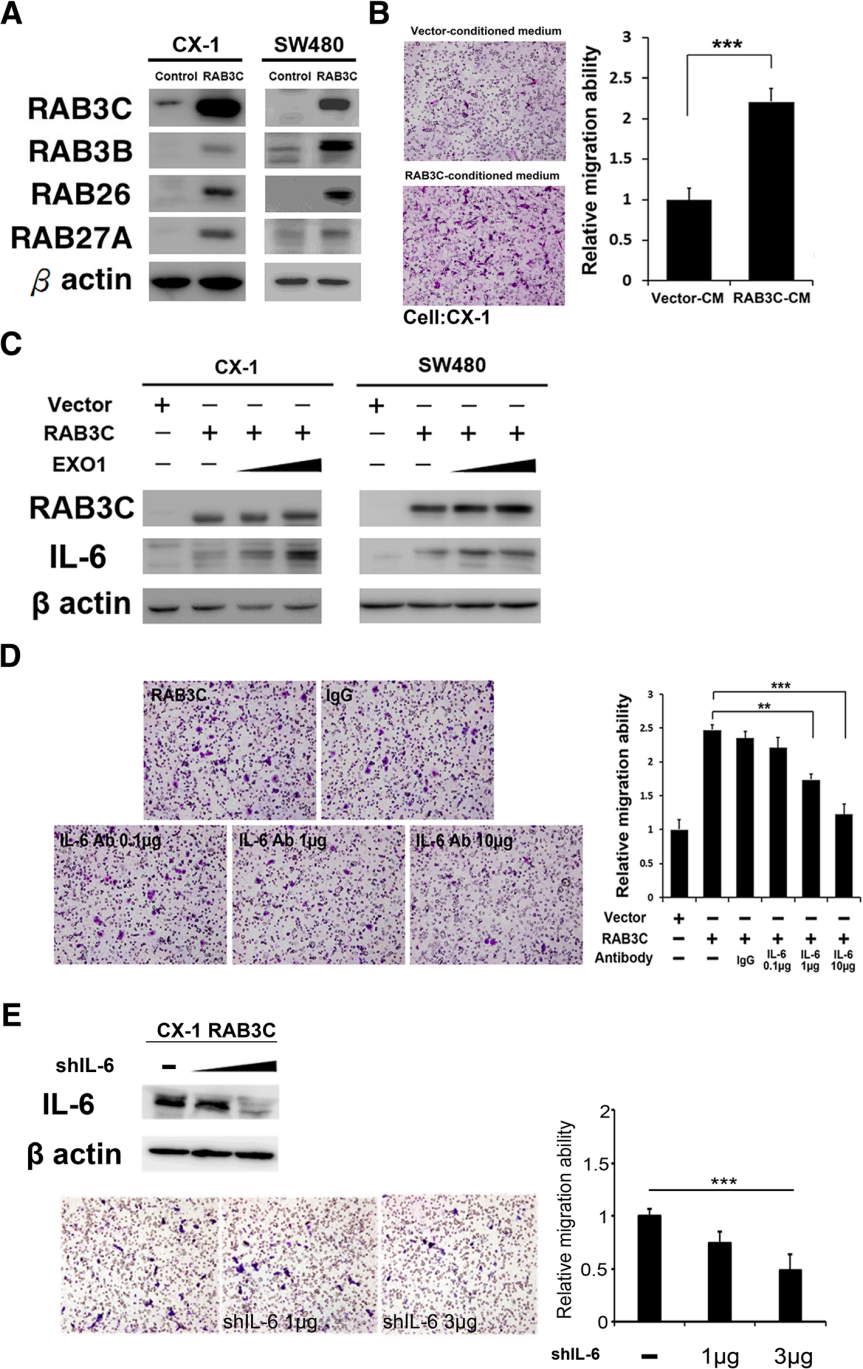
RAB3C overexpression increases exocytosis of colon cancer cells and promotes metastasis through IL-6 secretion (**a**) Western blot analysis of RAB3C, RAB3B, RAB26, RAB27A and β-actin protein expression with or without RAB3C overexpression in CX-1 and SW480 cells. **b** Migration ability of CX-1 cells induced by culture medium from RAB3C-overexpressing or control cells. **c** Western blot analysis of RAB3C, IL-6 and β-actin protein expression after dose-dependent EXO-1 treatment with or without RAB3C overexpression in CX-1 and SW480 cells. **d** Migration ability after IL-6 antibody treatment was dose-dependent in a RAB3C model. **e** Western blot analysis of IL-6 and β-actin protein expression and the migration ability of CX-1 cells after IL-6 dose-dependent knockdown and RAB3C-overexpression

On the basis of the finding that RAB3C-overexpressing cells exhibited increased cytokine expression, we further studied the role of RAB3C in exocytosis. In two colon cancer cell lines, we observed upregulation of other exocytic RABs including RAB3B, RAB26, and RAB27A as a result of RAB3C overexpression (Fig. 5a). Furthermore, conditioned medium was used in a Transwell migration assay to confirm whether the substances secreted by RAB3C overexpressing cells are the main mechanism through which RAB3C promotes metastasis. In the Transwell migration assay, the conditioned medium of RAB3C-overexpressing cells in CX-1 cells (Fig. 5b) and SW48 cells (Additional file 3: Figure S2A) and vector control cells were loaded in the lower part of the transwell device, and corresponding parental cells in serum-free medium were loaded in the upper portion of the Transwell device. The significant effect of the RAB3C overexpressing cell-conditioned medium on the migration ability of parental colon cancer cells indicated that the metastasis-promoting role of RAB3C was exocytosis dependent (Fig. 5b). In addition, Rab3C overexpressions in CX-1 and SW-48 cells increased the levels of IL-6 in conditioned mediums (Additional file 3: Figure S2B). There was also a gradient increase in the intracellular IL-6 level in RAB3C-overexpressing cells after treatment with 50 μM and 100 μM EXO1, an exocytosis inhibitor, as well as the finding that blocking IL-6 with IL-6 antibody added to the maintenance medium significantly decreased the migration ability of RAB3C-overexpressing cells in a dose-dependent manner, indicated that RAB3C regulates cancer metastasis through IL-6 exocytosis (Fig. 5c and d). We observed that IL-6 knockdown decreased the RAB3C-enhanced migration/invasion ability of colon cancer cells, thus indicating a critical role of the RAB3C-IL-6 axis in promoting the metastatic potential of colon cancer cells (Fig. 5e).

### RAB3C promotes colon cancer metastasis through IL-6 secretion and increased phosphorylation of STAT3

**Fig. 6:**
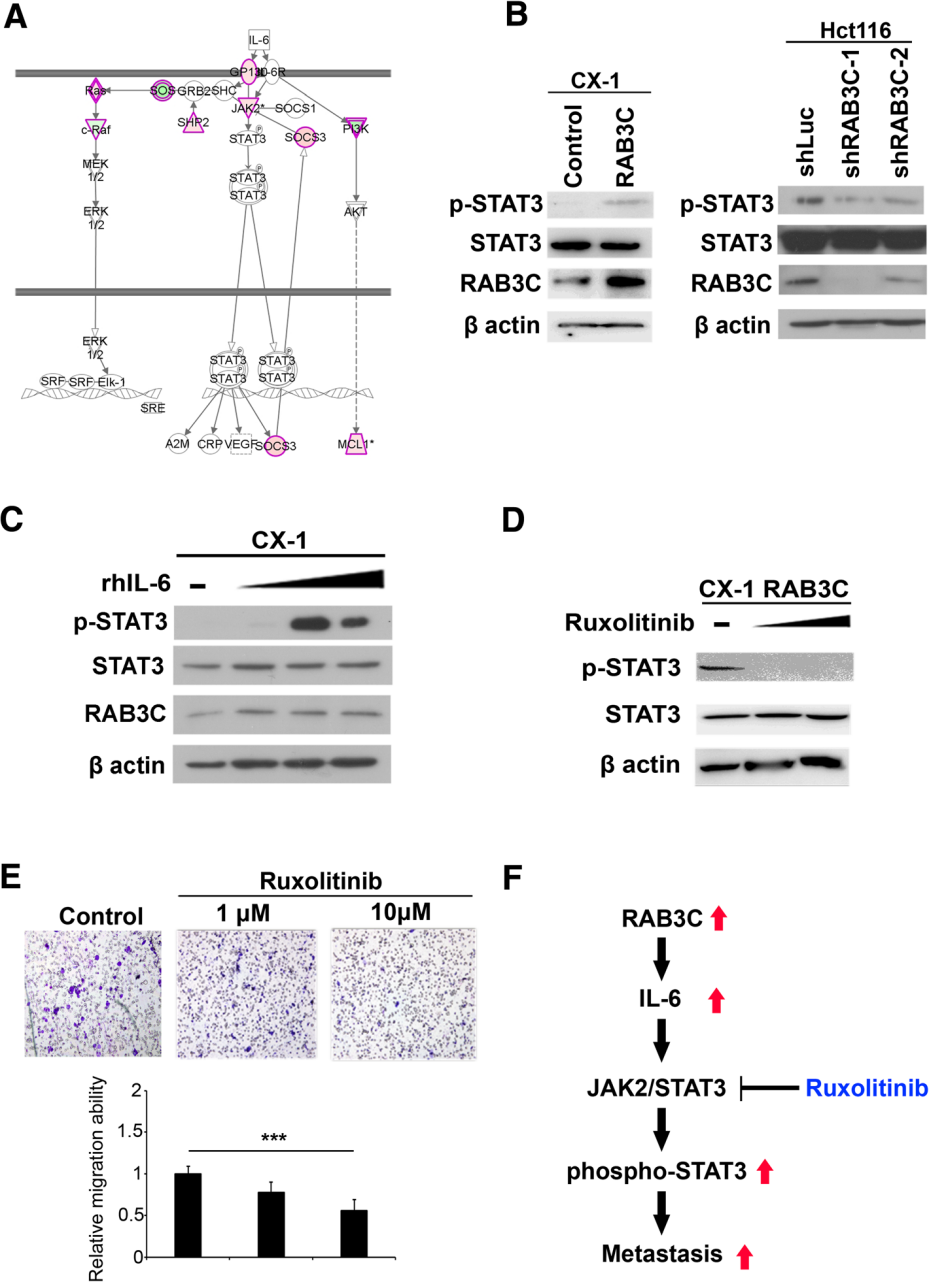
RAB3C enhanced STAT3 phosphorylation through IL-6, thereby promoting colon cancer metastasis (**a**) IL-6 signaling and the downstream pathway in RAB3C-overexpressing cells. Red indicates upregulation, and green indicates inhibition in the RAB3C-based microarray data. **b** Western blot analysis of phospho-STAT3, STAT3, RAB3C and β-actin protein expression with or without RAB3C-overexpression in CX-1 cells. **c** Western blot analysis of phospho-STAT3, STAT3, and β-actin protein expression with and without recombinant human IL-6 treatment. **d** Western blot analysis of phospho-STAT3, STAT3, and β-actin protein expression with or without Ruxolitinib treatment in a CX-1 RAB3C model. **e** Effect of different doses of Ruxolitinib on the migration ability of CX-1 cells. **f** Proposed model of the RAB3C/IL-6 axis in colon cancer progression

To further confirm the role of the RAB3C-IL-6 axis in colon cancer metastasis, we analyzed the canonical pathway of IL-6 by examining previous data. The results showed that JAK2 and SOCS3 were upregulated in RAB3C-overexpressing cells (Fig. 6a). Previous studies have shown that IL-6 induces JAK2 activation and STAT3 activation via Tyr705 phosphorylation. Thus, we detected the expression levels of total and phosphorylated STAT3. We found that STAT3 phosphorylation was increased after ectopic expression of RAB3C in CX-1 cells but was decreased after RAB3C knockdown (Fig. 6b). We next determined whether IL-6 might promote STAT3 phosphorylation in the absence of RAB3C overexpression in cells, by using recombinant IL-6 protein. We found that IL-6 directly triggered the phosphorylation of STAT3 in a dose-dependent manner in CX-1 cells (Fig. 6c). Moreover, treatment of RAB3C-overexpressing CX-1 cells with Ruxolitinib, a JAK2-specific inhibitor, clearly blocked the phosphorylation of STAT3. Our results showed that Ruxolitinib decreased the migration ability and the level of phosphorylated STAT3 in RAB3C-overexpressing CX-1 cells in a dose-dependent manner (Fig. 6d and e). On the basis of the evidence presented above, we propose that RAB3C overexpression in colon cancer cells induces IL-6 exocytosis, thereby triggering JAK2 activation and STAT3 phosphorylation and inducing cancer cell metastasis (Fig. 6f).

## Discussion

In this study, the high expression of RAB3C in colorectal cancer tissue compared with that in normal colonic mucosal tissue provided strong evidence allowing us to determine its value as a prognostic indicator. Survival analyses showed that patients with high RAB3C expression had poor overall and disease-free survival, and RAB3C overexpression remained an independent prognostic factor in multivariate analyses. Moreover, high RAB3C expression was significantly correlated with distant metastasis. RAB3C overexpression was also confirmed to increase the migration and invasion ability of colon cancer cells and the number of metastatic nodules in animal models. Knowledge-based pathway analysis using RNA microarray and proteomics data analysis revealed that the IL-6 pathway is the major signaling pathway involved in RAB3C’s effects. The gradient decrease in the migration ability induced by RAB3C-overexpressing cell-conditioned medium by blocking of IL-6 further indicated that promotion of metastasis by RAB3C depends on IL-6 secretion. Together, these results indicated that the RAB3C protein plays a critical role in tumor progression, invasion, and metastasis through IL-6 exocytosis.

The majority of research related to RAB3 has focused on its normal physiological functions, whereas relatively little is known about the role of RAB3 in tumorigenesis. Among 4 highly homologous isoforms of RAB3, their subcellular targets and functional roles have been proposed to be distinct because of differences in their N- and C-terminal domains [31, 32]. RAB3B, a key exocytosis regulator in anterior pituitary cells, has been demonstrated by immunohistochemistry staining to be overexpressed in pituitary adenoma [33, 34]. RAB3D, which is predominantly expressed in non-neuronal cells such as adipocytes and various exocrine glands, has recently been studied in breast cancer. However, there was no correlation between tumor progression and the presence of endogenous RAB3D mRNA and protein [24]. The metastasis-promoting ability of RAB3C in colorectal cancer in the present study underscored the importance of conducting more research to elucidate whether other RAB3 isoforms and other exocytic RABs also participate in and coordinately regulate exocytosis, thereby leading to tumor metastasis.

RAB3 has been found to regulate the final steps of exocytosis and function as a gate-keeper of late stage exocytosis [35]. Exocytosis is a critical factor in the adaptation of cancer cells to the challenging environment encountered during invasion and metastasis. Cancer cell exocytosis plays an important role in liberating growth factors into the microenvironment, thus facilitating invasive the growth of tumors. The importance of RABs in this process has been illustrated by two recent studies of RAB27 in breast cancer. Overexpression of RAB27A has been shown to enhance tumor invasion and metastasis in breast cancer cell lines through secretion of insulin-like factor-II (IGF-II), which in turn modulates many important tumor progression markers including p16, vascular endothelial growth factor, cathepsin D, cyclin D1, matrix metalloproteinase-9, and urokinase-type plasminogen activator [25]. In another study, heat-shock protein 90α has been identified in RAB27B-secretory vesicles as a key pro-invasive growth regulator inducing activation of matrix metalloproteinase-2 in breast cancer [24]. Moreover, this study has also revealed a correlation between RAB27B and poor differentiation and lymph node metastasis in ER-positive breast cancer.

Our research is the first study focused on the role and the function of RAB3C in cancer. In the present study, we found that IL-6 secretion is the major mechanism by which RAB3C induces cancer metastasis. IL-6 has been reported to induce tumor progression, especially metastasis, in various cancer types and is also considered to be a potential therapeutic target [36, 37]. In colon cancer, IL-6 participates in almost every step of cancer progression, including tumor initiation, proliferation, migration, and angiogenesis [38], and IL-6 expression has been confirmed to be correlated with poor prognosis [39]. IL-6 is generally known to be secreted by tumor-associated fibroblasts and to create an environmental niche for cancer progression [38, 40]. However, increasing evidence shows that tumor cell-secreted IL-6 also promotes tumorigenesis through autocrine regulation [41, 42]. In our study, we found that IL-6 secreted by colon cancer cells modulates tumor metastasis. Our study is the first to reveal the relationship between exocytic RABs and cytokine secretion, and it further solidifies the role of RAB-regulated IL-6 autocrine signaling in cancer progression.

In addition, secretory RABs control exosome secretion, thus facilitating angiogenesis, degradation of the extracellular matrix, and creation of an immune-privileged environment for cancer cells [43, 44]. Cancer progression markers, including molecules related to metastasis processes and signaling transduction and some lipid raft-associated proteins, have been isolated from metastatic colon cancer-derived exosomes [45]. In addition, the level of circulating exosomes has also been reported to be an indicator of colon cancer prognosis [30]. Exosomes also affect chemoresistance and chemosensitivity by modulating drug efflux mechanisms against cytotoxic drugs such as cisplatin and microtubule stability targeted by drugs such as taxanes [46, 47]. The strong effects of RAB3C expression on disease-free survival and tumor recurrence in the present study may be attributed to treatment resistance modulated by RAB3C. However, whether and how RAB3C-regulated exocytosis has a direct effect on chemoresistance needs further exploration. Furthermore, recent research on blocking exosome liberation by interfering with exocytic RABs also provided new insights in studying chemoresistance mechanisms [44].

In conclusion, increased RAB3C expression is correlated with poor prognosis and distant metastasis in colorectal cancer patients and regulates exocytosis and IL-6 secretion. Moreover, its further activation of the JAK2-STAT3 signaling pathway may be essential for tumor invasiveness and metastasis. Our study not only suggests a new direction for studies focused on deciphering the relationship between exocytic RABs and cancer progression but also reveals that the RAB3C-IL6-STAT3 axis may serve as a target for prognostic prediction and future therapeutic intervention with drugs such as Ruxolitinib.

## Conclusions

This study demonstrated that RAB3C overexpression is associated with tumor metastasis and poor prognosis in colorectal cancer, through modulating exocytosis of IL-6 in cancer cells, thus leading to activation of the IL6-JAK2-STAT3 pathway. Furthermore, suppression of STAT3 phosphorylation in the RAB3C-IL-6-STAT3 axis by Ruxolitinib may offer new hope for physicians to combat metastatic colon cancers.

## Additional files

Additional file 1: Table S1. Correlation of clinicopathological features of colorectal cancer patients and the RAB3C tumor expression. (DOCX 2201 kb)

Additional file 2: Figure S1. High RAB3C expression is an independent indicator in colorectal cancer patients. (A) the box plot shows that higher RAB3C expression was correlated with a poor overall survival rate in patients in the GSE17536, (n = 177) from the SurvExpress database (*P* = 0.015). (B) Heatmap indicates RABs family mRNA level correlated with grade and pathological stage in the clinicopathological analysis by the Oncomine online tool. (TIFF 32000 kb)

Additional file 3: Figure S2. RAB3C overexpression increases exocytosis of colon cancer cells and promotes metastasis through IL-6 secretion. (A) The significant effect of RAB3C overexpressing cell-conditioned medium on the migration ability of parental colon cancer cells indicate that the metastasis-promoting role of RAB3C is exocytosis dependent. (B) Relative IL-6 activity in conditioned medium of CX-1 cells and SW48 cells with or without the exogenous RAB3C gene. The data were the average of three independent experiments and are presented as the mean ± SEM. The significance of the difference was analyzed using the nonparametric Mann-Whitney *U* test. (TIFF 28902 kb)

## Abbreviations

EXO1: Exonuclease1, 2-(4-fluorobenzoylamino)-benzoic acid methyl ester
IL-6: Inteleukin-6
IPA: Ingenuity pathway analysis
RAB: Ras-related protein
STAT3: Signal transducer and activator of transcription 3
