# Supplementary material for: Secretory RAB GTPase 3C modulates IL6-STAT3 pathway to promote colon cancer metastasis and is associated with poor prognosis
Source: Mol Cancer. 2017 Aug 7;16:135. doi: 10.1186/s12943-017-0687-7 (PMC5547507; doi:10.1186/s12943-017-0687-7)

Additional file 1: **Table S1**: Correlation of clinicopathological features of colorectal cancer patients and the RAB3C tumor expression

| Clinicopathological feature | n | Rab3C expression, n (%) | | *P* |
| --- | --- | --- | --- | --- |
|  |  | Low  (n=90) | High  (n=125) |  |
| Age |  |  |  |  |
| <65 y | 70 | 32 (45.7) | 38 (54.3) | 0.258 |
| ≧65 y | 145 | 58 (40.0) | 87 (60.0) |  |
| Gender |  |  |  |  |
| Male | 120 | 49 (40.8) | 71 (59.2) | 0.419 |
| Female | 95 | 41 (43.2) | 54 (56.8) |  |
| Tumor location |  |  |  |  |
| Right colon | 37 | 12 (32.4) | 25 (67.6) | 0.449 |
| Transverse colon | 27 | 13 (48.1) | 14 (51.9) |  |
| Descending colon | 17 | 9 (52.9) | 8 (47.1) |  |
| Rectosigmoid colon | 134 | 56 (41.8) | 78 (58.2) |  |
| T stage |  |  |  |  |
| T1+T2 | 28 | 15 (53.6) | 13 (46.4) | 0.127 |
| T3+T4 | 187 | 75 (40.1) | 112 (59.9) |  |
| N stage |  |  |  |  |
| N0 | 99 | 46 (46.5) | 53 (53.5) | 0.130 |
| N1+N2 | 116 | 44 (37.9) | 72 (62.1) |  |
| M stage |  |  |  |  |
| M0 | 179 | 84 (46.9) | 95 (53.1) | <0.001 |
| M1 | 36 | 6 (16.7) | 30 (83.3) |  |
| Pathological stage |  |  |  |  |
| I+II | 88 | 44 (50.0) | 44 (50.0) | 0.044 |
| III+IV | 127 | 46 (36.2) | 81 (63.8) |  |
| Vascular invasion |  |  |  |  |
| No | 98 | 46 (46.9) | 52 (53.1) | 0.107 |
| Yes | 117 | 44 (37.6) | 73 (62.4) |  |
| Perineural invasion |  |  |  |  |
| No | 164 | 73 (44.5) | 91 (55.5) | 0.105 |
| Yes | 51 | 17 (33.3) | 34 (66.7) |  |
| Tumor histology |  |  |  |  |
| Nonmucinous | 198 | 86 (43.4) | 112 (56.6) | 0.088 |
| Mucinous | 17 | 4 (23.5) | 13 (76.5) |  |
| Recurrence |  |  |  |  |
| No | 141 | 68 (48.2) | 73 (51.8) | 0.006 |
| Yes | 74 | 22 (29.7) | 52 (70.3) |  |

**Supplementary Figure**


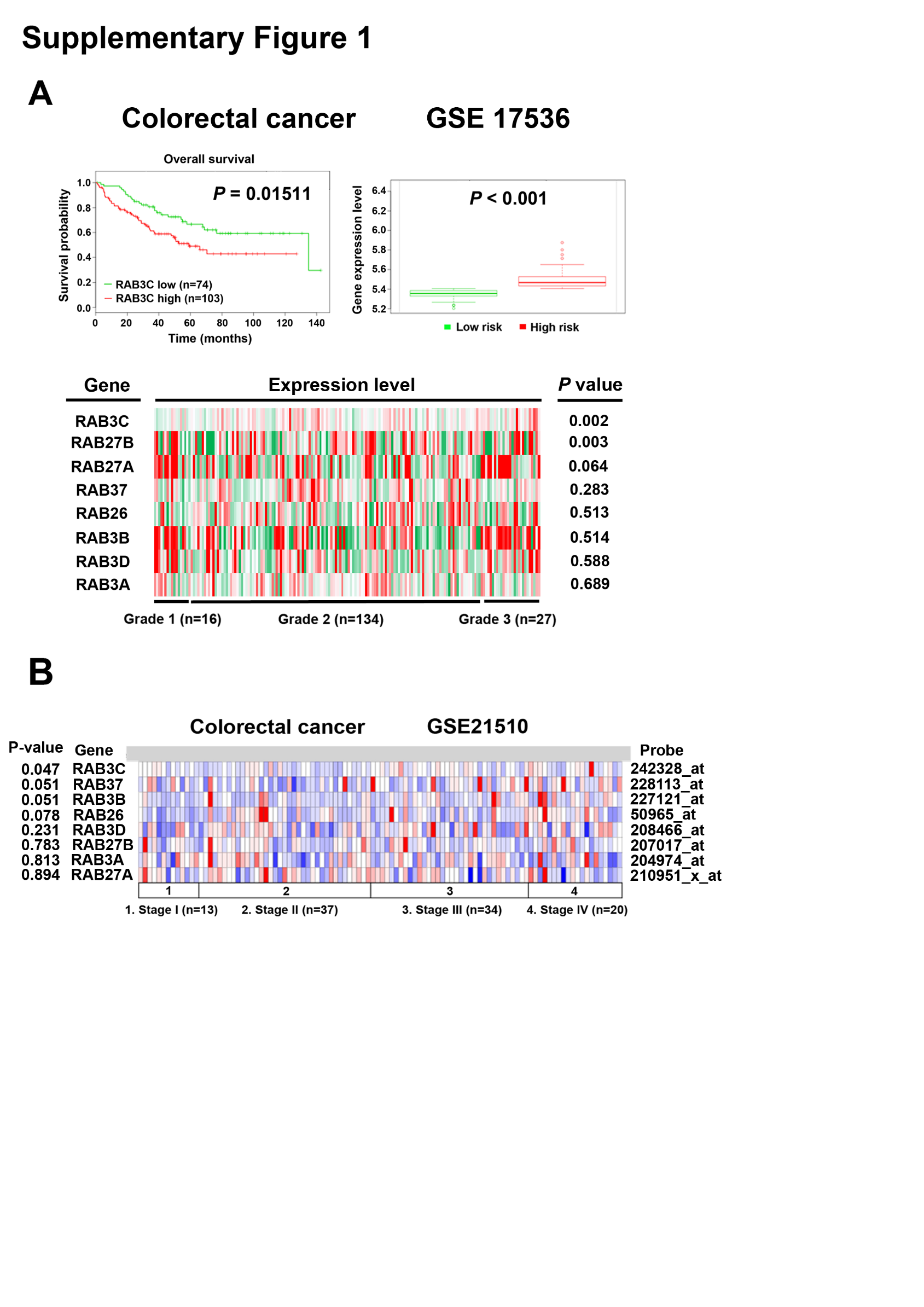


**Supplement Figure 2**


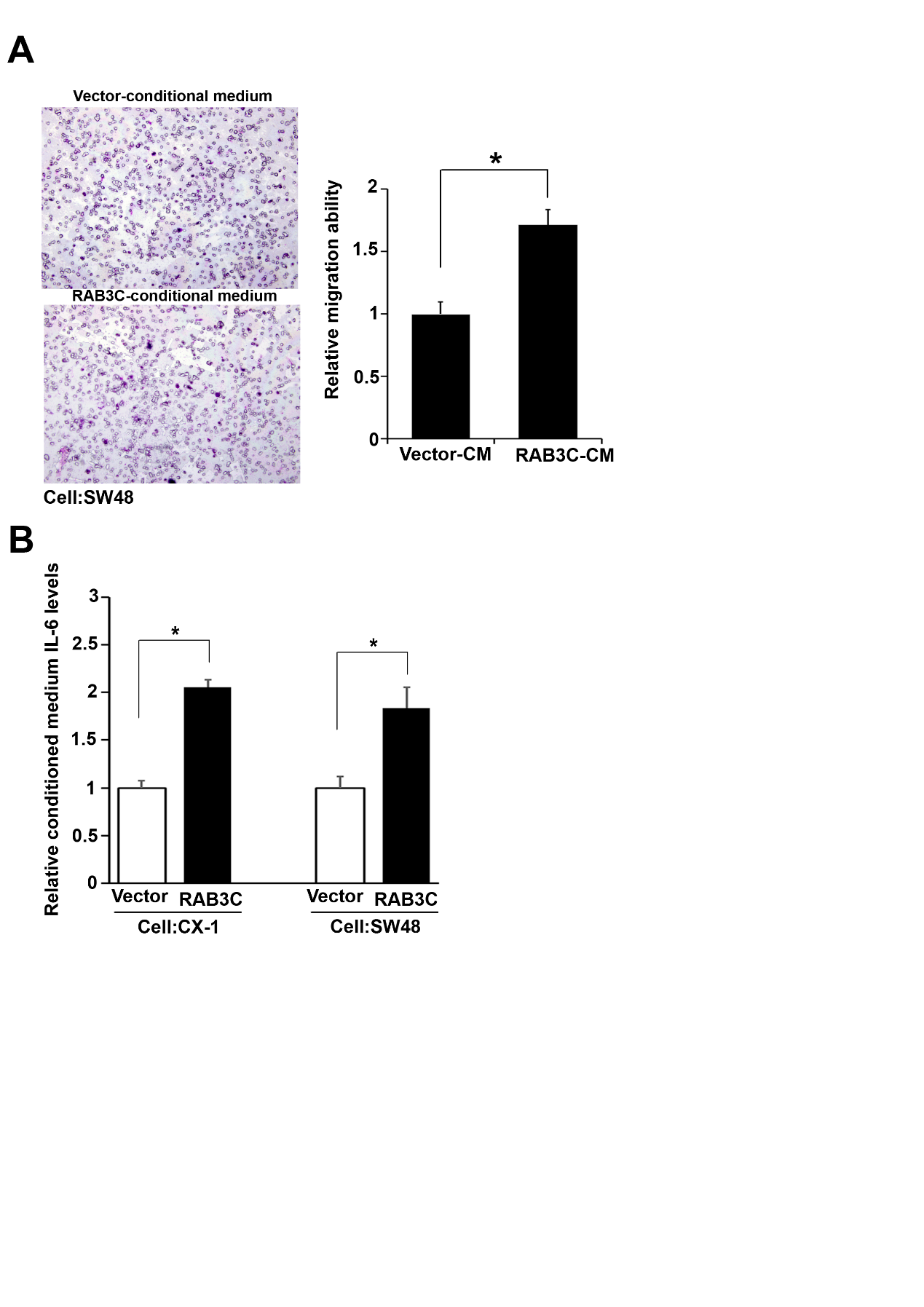

Supplement: Supplementary file 1 — Correlation of clinicopathological features of colorectal cancer patients and the RAB3C tumor expression. (DOCX 2201 kb) [file 12943_2017_687_MOESM1_ESM.docx]
