# Supplementary figures and images for: Secretory RAB GTPase 3C modulates IL6-STAT3 pathway to promote colon cancer metastasis and is associated with poor prognosis
Source: Mol Cancer. 2017 Aug 7;16:135. doi: 10.1186/s12943-017-0687-7 (PMC5547507; doi:10.1186/s12943-017-0687-7)

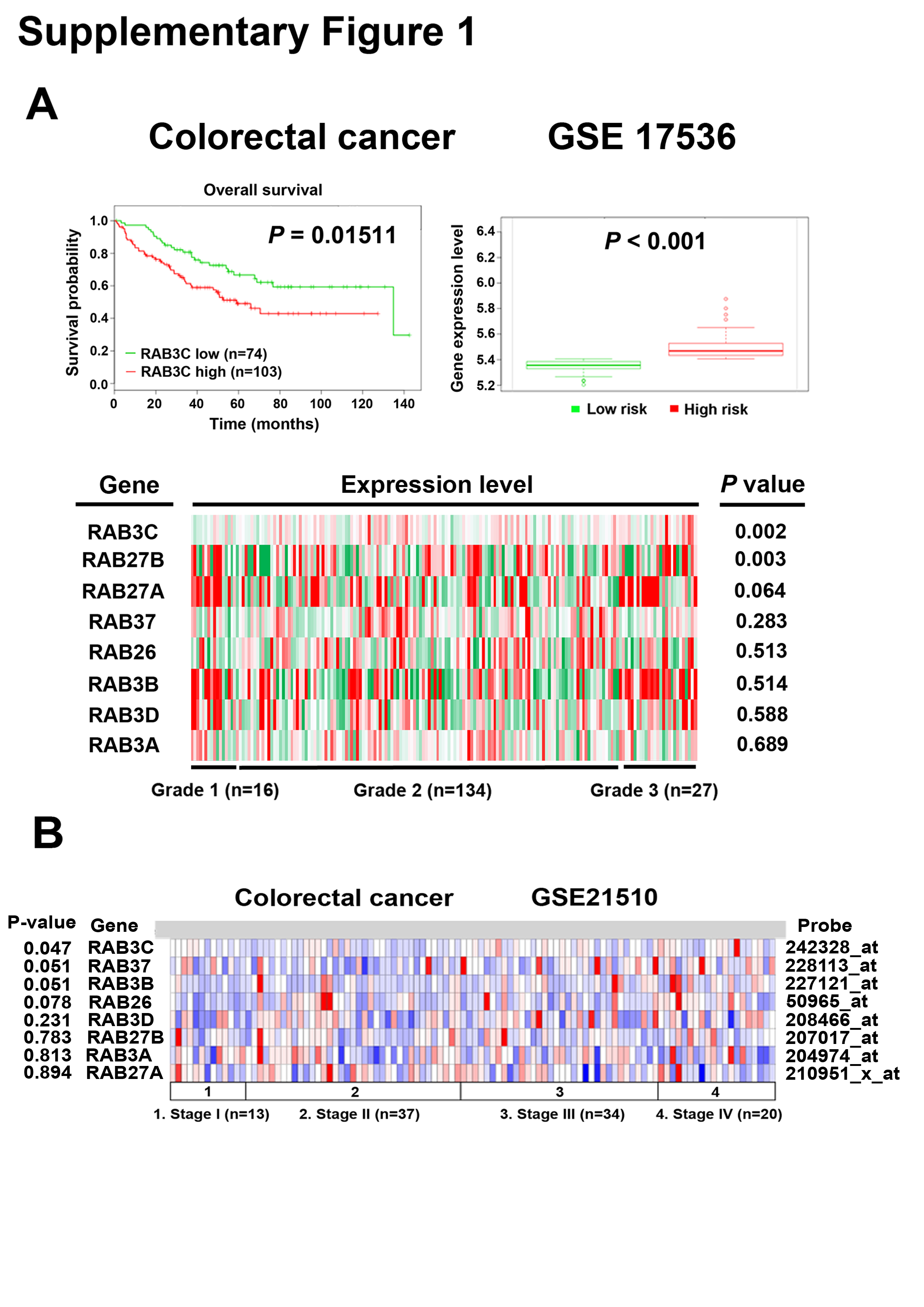

Supplement: Supplementary file 2 — High RAB3C expression is an independent indicator in colorectal cancer patients. (A) the box plot shows that higher RAB3C expression was correlated with a poor overall survival rate in patients in the GSE17536, (n = 177) from the SurvExpress database (P = 0.015). (B) Heatmap indicates RABs family mRNA level correlated with grade and pathological stage in the clinicopathological analysis by the Oncomine online tool. (TIFF 32000 kb) [file 12943_2017_687_MOESM2_ESM.tif]

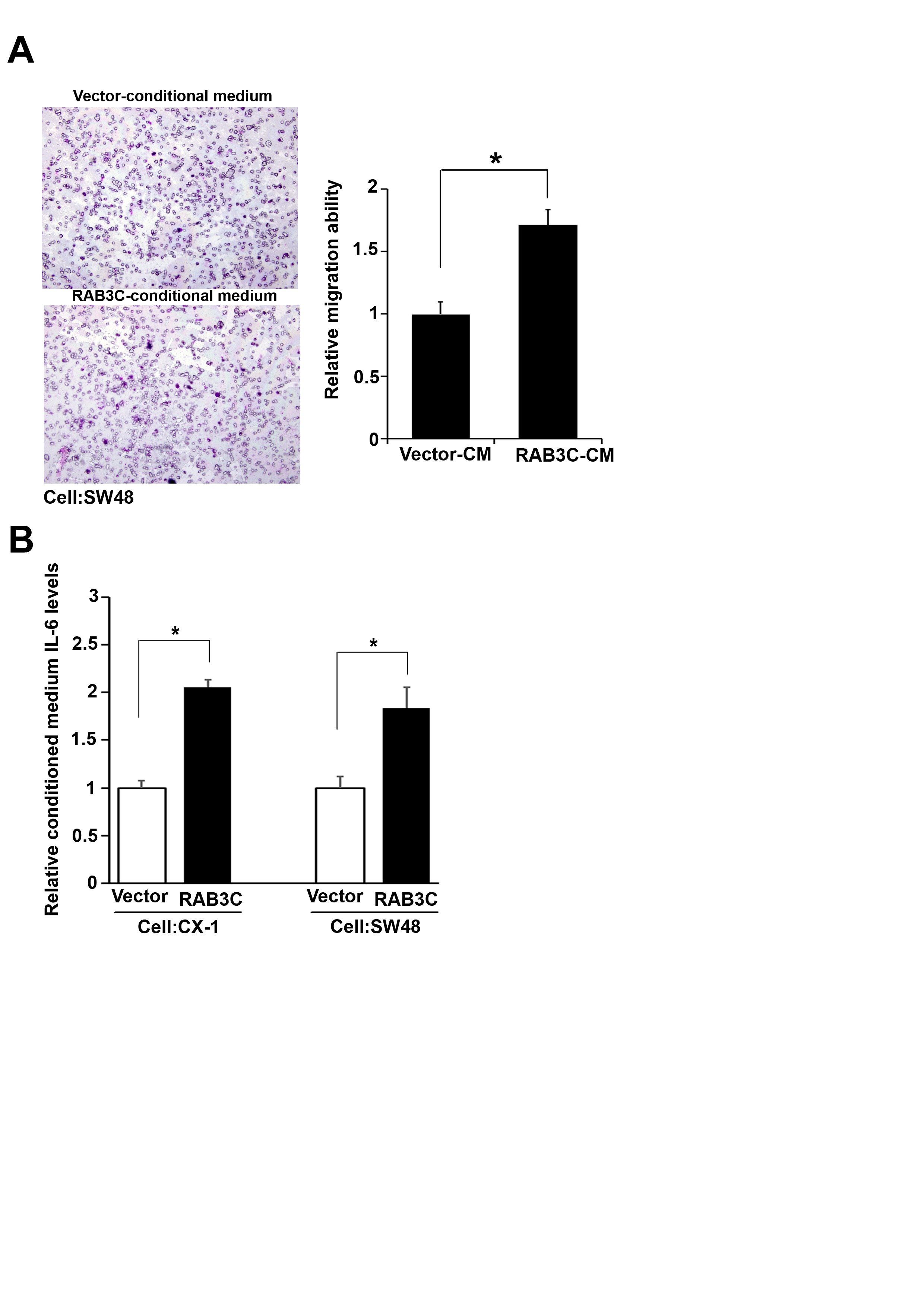

Supplement: Supplementary file 3 — RAB3C overexpression increases exocytosis of colon cancer cells and promotes metastasis through IL-6 secretion. (A) The significant effect of RAB3C overexpressing cell-conditioned medium on the migration ability of parental colon cancer cells indicate that the metastasis-promoting role of RAB3C is exocytosis dependent. (B) Relative IL-6 activity in conditioned medium of CX-1 cells and SW48 cells with or without the exogenous RAB3C gene. The data were the average of three independent experiments and are presented as the mean ± SEM. The significance of the difference was analyzed using the nonparametric Mann-Whitney U test. (TIFF 28902 kb) [file 12943_2017_687_MOESM3_ESM.tif]
